# Aquatic microfauna alter larval food resources and affect development and biomass of West Nile and Saint Louis encephalitis vector *Culex nigripalpus* (Diptera: Culicidae)

**DOI:** 10.1002/ece3.2947

**Published:** 2017-04-09

**Authors:** Dagne Duguma, Michael G. Kaufman, Arthur B. Simas Domingos

**Affiliations:** ^1^ Florida Medical Entomology LaboratoryUniversity of Florida/IFASVero BeachFLUSA; ^2^ Department of EntomologyMichigan State UniversityEast LansingMIUSA

**Keywords:** bacteria, ciliate protist, *Culex* mosquito, disease vectors, food web, *Habrotrocha rosa*, rotifers, trophic interactions, vector control

## Abstract

Ciliate protists and rotifers are ubiquitous in aquatic habitats and can comprise a significant portion of the microbial food resources available to larval mosquitoes, often showing substantial declines in abundance in the presence of mosquito larvae. This top‐down regulation of protists is reported to be strong for mosquitoes inhabiting small aquatic containers such as pitcher plants or tree holes, but the nature of these interactions with larval mosquitoes developing in other aquatic habitats is poorly understood. We examined the effects of these two microbial groups on lower trophic level microbial food resources, such as bacteria, small flagellates, and organic particles, in the water column, and on *Culex* larval development and adult production. In three independent laboratory experiments using two microeukaryote species (one ciliate protist and one rotifer) acquired from field larval mosquito habitats and cultured in the laboratory, we determined the effects of *Culex nigripalpus* larval grazing on water column microbial dynamics, while simultaneously monitoring larval growth and development. The results revealed previously unknown interactions that were different from the top‐down regulation of microbial groups by mosquito larvae in other systems. Both ciliates and rotifers, singly or in combination, altered other microbial populations and inhibited mosquito growth. It is likely that these microeukaryotes, instead of serving as food resources, competed with early instar mosquito larvae for microbes such as small flagellates and bacteria in a density‐dependent manner. These findings help our understanding of the basic larval biology of *Culex* mosquitoes, variation in mosquito production among various larval habitats, and may have implications for existing vector control strategies and for developing novel microbial‐based control methods.

## 1 Introduction

Heterotrophic microorganisms such as protists and rotifers are ubiquitous in aquatic mosquito larval habitats and are important components of the microbial food web that is a primary nutritional resource for larvae, particularly in container‐type larval mosquito habitats (Addicott, 1974; Arndt, 1993; Blaustein & Chase, 2007; Kaufman, Goodfriend, Kohler‐Garrigan, Walker, & Klug, 2002; Kneitel, 2007; Stoecker & Capuzzo, 1990; Walker, Kaufman, & Merritt, 2010; Wallace & Smith, 2013; Wallace & Snell, 2010). The interactions of these microeukaryotes with mosquito larvae mainly as a top predator have been well studied in phytotelmata such as in pitcher plants *Sarracenia purpurea* L. and in tree hole systems (Addicott, 1974; Cochran‐Stafira & von Ende, 1998; Eisenberg, Washburn, & Schreiber, 2000; Hoekman, 2007, 2011; Walker, Kaufman, & Merritt, 2010; Washburn, 1995). However, this knowledge is largely limited to species of *Aedes* or *Wyeomyia* mosquitoes inhabiting small container mosquito habitats, and less is known about the interaction of these microbes with other mosquito taxa.

Both protists and rotifers primarily feed on bacteria (Gatesoupe, 1991; Pernthaler, 2005; Wallace & Smith, 2013), whereas mosquito larvae are considered omnivores feeding on varied microorganisms including protists and rotifers, as well as bacteria, fungi, and microalgae (Merritt, Dadd, & Walker, 1992). Protists and rotifers are considered intermediate consumers in aquatic food webs, including in mosquito larval habitats, and are known to transfer bacterial biomass to larger consumers including mosquito larvae (Banerji et al., 2015; Kneitel, 2007; Pace & Orcutt, 1981; Pernthaler, 2005; Stoecker & Capuzzo, 1990). Mosquito larvae can be major predators of microorganisms in the absence of other large predators especially during early habitat succession of larval aquatic habitats (Batzer & Wissinger, 1996; Lawler & Dritz, 2005; Peck & Walton, 2008). A previous study that removed *Culex* mosquito larvae from the water column using a larvicide detected trophic cascade effects (i.e., reduction in abundance of photosynthetic microbes and increase in bacterial diversity) in the water column, but that study did not address whether population dynamics of heterotrophic microeukaryotes were also altered as a result of reduced larval mosquito abundance (Duguma et al., 2015). This work indicated release of predation pressure from mosquitoes might have increased the abundance of microeukaryotes, which in turn might have influenced water column bacterial community composition. In another study, a proliferation of several genera of protists following elimination of floodwater *Aedes* mosquito larvae using *Bti* larvicide was observed, suggesting a strong top‐down regulation of the protist population by *Aedes* mosquito larvae (Östman, Lundström, & Persson Vinnersten, 2008). However, Skiff and Yee (2015) found that the effect of feeding several species of protists to larvae on the survival of *Culex quinquefasciatus, Culex coronator*, and *Aedes albopictus* mosquitoes was negligible, suggesting that top‐down regulation may not be meaningful to larval nutrition. Investigating interactions of these microorganisms with mosquito larvae are critical to understand trophic interactions and potentially unintended ecological consequences of larval mosquito control using pesticides on aquatic ecosystems. Because particulate ingestion varies among different species of mosquito larvae and different larval stages (e.g., Dahl, Sahlen, Grawe, Johannisson, & Amneus, 1993), top‐down or bottom‐up population regulation hypotheses may not apply to some mosquito species.

Ciliate protists and rotifers are important filter feeders in the water column and on substrates (Blaustein & Chase, 2007; Pernthaler, 2005; Sherr & Sherr, 2002; Stoecker & Capuzzo, 1990), but the effects of these microorganisms on controphic filter feeders such as mosquitoes and on lower trophic level microorganisms such as bacteria in larval mosquito habitats are not well explored (Blaustein & Chase, 2007; Pernthaler, 2005). Although much previous work suggests that protists and rotifers are readily consumed by larval mosquitoes, it is possible that some microeukaryotes and mosquito larvae compete for particles in the bacteria size ranges. Our initial hypothesis was that additions of protists and/or rotifers would provide more food sources for developing larvae, as in the case of most planktonic filter feeders in aquatic systems (Porter, Pace, & Battey, 1979; Sanders & Wickman, 1993). We tested this by measuring larval development, adult emergence, and adult biomass of *Culex nigripalpus* in laboratory microcosms supplemented with the addition of ciliate and rotifer species. *Cx. nigripalpus* Theobald is an important vector of Saint Louis encephalitis virus and other pathogens in southeastern US (Day & Stark, 2000; Sardelis, Turell, Dohm, & O'Guinn, 2001), and is primarily a water column filter feeder in the larval stage. We also hypothesized that these two microbial groups alter the population dynamics of bacteria and other microeukaryotes in the water column. To test this hypothesis, we characterized the abundance of lower trophic level microorganisms such as bacteria and similarly sized microbes in the presence or absence of a ciliate and rotifer species isolated from larval *Cx. nigripalpus* habitats.

## 2 Material and Methods

### 2.1 Monocultures of two microeukaryotes

A bdelloid rotifer (*Habrotrocha rosa* Donner) and a ciliate protist (*Paramecium* sp.) derived from an outdoor aquatic mosquito research facility at the Florida Medical Entomology Laboratory (FMEL) were established in the laboratory in April 2015. These microorganisms were originally collected from aquatic mesocosms colonized by *Culex* mosquitoes including *Cx. nigripalpus*. The ciliate was 218 ± 7 μm (mean, SE; *n* = 6) long and identified to *Paramecium* sp. using keys (Foissner & Berger, 1996), whereas *H. rosa* species was 267 ± 17 μm (*n* = 6) long (Figure S1). The cultures of these two species were maintained on 0.1% (W:V) timothy hay extract at room temperature. The extract was made by adding 20 g of dried hay in 2 L of distilled water and boiling for 30 min. The liquid suspension was then cooled down to room temperature, and coarse materials above 53‐μm diameter were removed using a 53‐μm (300 nylon mesh) screen. Up to three wheat seeds were added to the medium for additional protein and lipid sources for bacteria or other microbial taxa.

### 2.2 Larval mosquitoes

Egg rafts of wild *Cx. nigripalpus* mosquitoes were collected from an outdoor aquatic mosquito research facility at the FMEL. Two livestock cattle tanks (mesocosms) were infused with 0.2 kg of alfalfa rabbit food in 300 L of well water. The design and features of these mesocosms were described in another study (Duguma, Hall, Smartt, & Neufeld, 2017). The infusion was incubated anaerobically by covering the mesocosms with tarps. Infusions made of hay are routinely used as oviposition attractants for many *Culex* species (Hazard, Mayer, & Savage, 1967). Egg rafts laid by *Culex* mosquitoes were collected 24 hr after uncovering mesocosms, rinsed twice with distilled water, and individually placed in wells of sterile tissue culture plates filled with distilled water and transported to the laboratory. The eggs were allowed to hatch in an environmental chamber at a temperature of 27°C. The first instar larvae hatched from the eggs were identified as *Cx. nigripalpus* using keys (Cutwa & O'Meara, 2006), and the larval instars from the different egg rafts were pooled in a 2‐L sterile pan filled with distilled water. From this pool, 30 or 50 first instars were taken and washed three times with distilled water and transferred into each of 1‐L amber, glass bottles for different experiments. Amber glass bottles were used to allow optimum conditions for microbial growth and protect microbes from photodegradation.

### 2.3 Microeukaryote treatments

Three independent laboratory experiments were conducted using hay culture as food for both microeukaryotes and mosquito larvae. In the first experiment, three microbial treatments [*Paramecium* sp. at a density of 247 ± 23 (mean, SE) protist per ml; *H. rosa* at 84 ± 6 rotifers per ml; and a combination of *H. rosa* and *Paramecium* sp. at 172 ± 6.5 cells/ml] were added to triplicate 1‐L amber glass containers to determine the effects of these microfauna on larval development and adult biomass of *Cx. nigripalpus*, and lower trophic level microbes including bacteria. These numbers of cells were achieved by adding 200 ml from each of the stock culture containing protists and rotifers to each of their respective treatments. Subsamples of 100 ml from each of *Paramecium* sp. and *H. rosa* stock cultures were combined for the combination of species treatment. The stock cultures were approximately 4 weeks old. In addition, 50 ml of previously prepared and refrigerated at a temperature of 6–8°C hay extract (10 days old) was added in to each of the containers for additional new nutrients. An untreated control consisted of 250 ml of hay extract and 250 ml of distilled water. Thirty 1st instar (24 hr old), *Cx. nigripalpus* larvae were added in to each of the containers.

In the second experiment, three treatments were used to assess whether *Paramecium* sp. dynamics, and feeding on lower trophic level organisms differ in the presence of *Culex* larvae. The treatments included a *Paramecium* sp. density of 29.6 ± 1.95 (mean and SE; *n* = 4) per ml and fifty 1st instar (< a day old) *Cx. nigripalpus*; a *Paramecium* sp. density of 29.6 ± 1.95 (mean and SE; *n* = 4) per ml without *Cx. nigripalpus*; and fifty 1st instar larvae of *Cx. nigripalpus* without *Paramecium* sp. Results of the first experiment indicated that we should increase the number of individual mosquitoes from thirty to fifty 1st instar larvae per replicate container in order to increase the levels of adult emergence from treated containers. This level of mosquito abundance is within the expected ranges of larval abundance obtained per standard mosquito dip sample of water in *Culex* mosquito larval habitats. For each treatment, *Paramecium* sp. and *Culex* larvae were added to a culture of 100 ml of a 4‐day‐old hay media diluted in 400 ml distilled water and replicated four times in amber glass containers. The hay medium was colonized by bacteria and small (3–5 μm, Estimated Spherical Diameter ESD) flagellates at the time treatments were applied.

In the final experiment, three density treatments (high = 40 ± 1.4 SE, medium = 16.7 ± 3, and low = 1.7 ± 1 protists per ml) of *Paramecium* sp., and an untreated control (i.e., without *Paramecium* sp.), and fifty 1st instars of *Cx. nigripalpus* larvae were added to a freshly prepared hay culture to determine whether the ciliate affects *Culex* larvae development and abundance of lower trophic microorganisms in a density‐dependent manner. Because we used a freshly prepared hay medium (i.e., 3 hr before the onset of the experiment) for this experiment, bacteria and other flagellates were not detected in the water at the time the experiment was initiated. The rotifer treatment was not used in the last two experiments due to an unforeseen decline in the population during winter. In this experiment, we attempted to create the very early likely succession pattern of microbes in the natural *Culex* mosquito larval environment, and to determine whether the *Culex* larvae would behave differently as early instars with lower abundances of bacteria and flagellates (i.e., consume ciliates as opposed to other microbial populations in previous experiments). Bacterial abundance available for mosquito larvae immediately after inundation/flooding might differ in abundance and diversity from that of later stages of aquatic habitat succession.

All experiments were conducted in amber glass bottles at 27°C at 12:12 hr light: dark cycle in an environmental chamber. The containers were rotated on the shelf in the environmental chamber every 24 hr.

### 2.4 Mosquito development and adult production

Containers were monitored daily for pupal development. Adults emerged from the containers were collected by placing the top part of a BioQuip mosquito breeder (BioQuip Inc., Rancho Dominguez, CA, USA) on top of the containers. Alternatively, we removed pupae using clean disposable pipets, and individually placed them in 20‐ml tubes in separate containers or cages for adult emergence. Emerged adults were sexed and individually placed in 1.5‐ml tubes and preserved at −20°C until biomass measurements were taken. In addition, development time to pupation and adult emergence were recorded. Adults were then oven‐dried at 51°C for 48 hr and weighed on microbalance (Orion Cahn C‐33 Microbalance; Thermo Fisher Scientific Inc, Cambridge, MA, USA).

### 2.5 Microbial (particle) dynamics

Microbial cells (organic particles) abundance and dynamic size ranging between 0.2 and 60 μm ESD in the water column were quantified using a Multisizer electronic counter 4e (Beckman Coulter Inc., Miami, FL, USA) according to Duguma et al. (2015). Briefly, a sample volume of 5‐ml culture was taken from each of the containers for microbial analyzes every day for the first three days, and then at 7 and 14 days after the initiation of the experiments. From these samples, cell (particle) counts were conducted in triplicate in 10 μl and 500 μl volumes of subsamples using the 10‐ and 100‐μm‐aperture tubes of the multisizer, respectively. For the 10‐μm aperture, a small subsample (1–2 ml) was filtered through a 5‐μm syringe filter to remove larger particles to avoid blockage by the 10‐μm‐aperture tube. The 10‐μm‐aperture tube was used to quantify particles (cells) ranging between 0.2 and 2.0 μm ESD that included the majority of bacterial cells, whereas the 100‐μm‐aperture tube was used to measure larger particles (cells) ranging between 2.0 and 60 μm ESD, which included some flagellates and other large bacterial cells.

An additional triplicate 0.1–0.5 ml subsamples were taken to monitor *H. rosa*,* Paramecium* sp., bacteria and other microbe population dynamics in the water using an inverted Leica (DM IL LED Fluo) microscope (Leica Microsystems Inc., Buffalo Grove, IL, USA). Bacteria and other small microbes in the water were visualized and photographed using a Leica DFC3000 G camera and Leica Application Suite software (Leica Microsystems Inc.).

### 2.6 Statistical analyzes

Data from the three experiments were analyzed independently. Mosquito data from the first experiment were analyzed with nonparametric Kruskal–Wallis tests (ANOVA on ranks) due to lack of adult emergence in most of the treated replicates. For the second and third experiments, mosquito data were analyzed with standard MANOVA methods, using log‐transformed averaged adult weights and emergence times, total adult weight, and total number of adults produced (per container in all cases). Univariate tests (ANOVA) were then performed on each dependent variable, and acceptable *p*‐value significance levels were adjusted with sequential Bonferroni correction (Rice, 1989). Further post hoc analyzes of differences between means in significant ANOVAs were done using the Tukey‐Kramer Highly significant difference (HSD) procedure.

Particle and microbial count data were averaged per replicate container, and log‐ and square root‐transformed, respectively, to achieve normality, and subjected to mixed model procedure to determine differences of particle and microbial abundance between treatments across dates during the larval development period. Significantly, different means among treatments were separated by Tukey‐Kramer's HSD test.

The relationships of microeukaryotes, particles, and mosquito parameters were examined with a Structural Equation Model (SEM) using PROC CALIS and the maximum likelihood method in SAS v 9.4 (SAS Institute Inc., Cary, NC, USA). The microeukaryotes and particle count data were averaged across sampling days per container, and then were square root‐and log‐transformed, respectively, to meet the normality assumptions of SEM. The analysis was restricted to experiments that yielded complete development of mosquito larvae (i.e., only containers from which adults emerged).

Unless stated otherwise, all other statistical analyzes were performed using JMP^®^ Pro 11 (SAS Inc., 2013). All graphs were made in Graphpad Prism v7 (GraphPad Software Inc., San Diego, CA, USA).

## 3 Results

### 3.1 Effects on larval development and adult biomass

Additions of the microeukaryotes had substantial negative effects on adult emergence and biomass. These effects differed somewhat depending on the experiment and levels of supplemented microeukaryotes.

#### 3.1.1 Mosquito production

**Figure 1:**
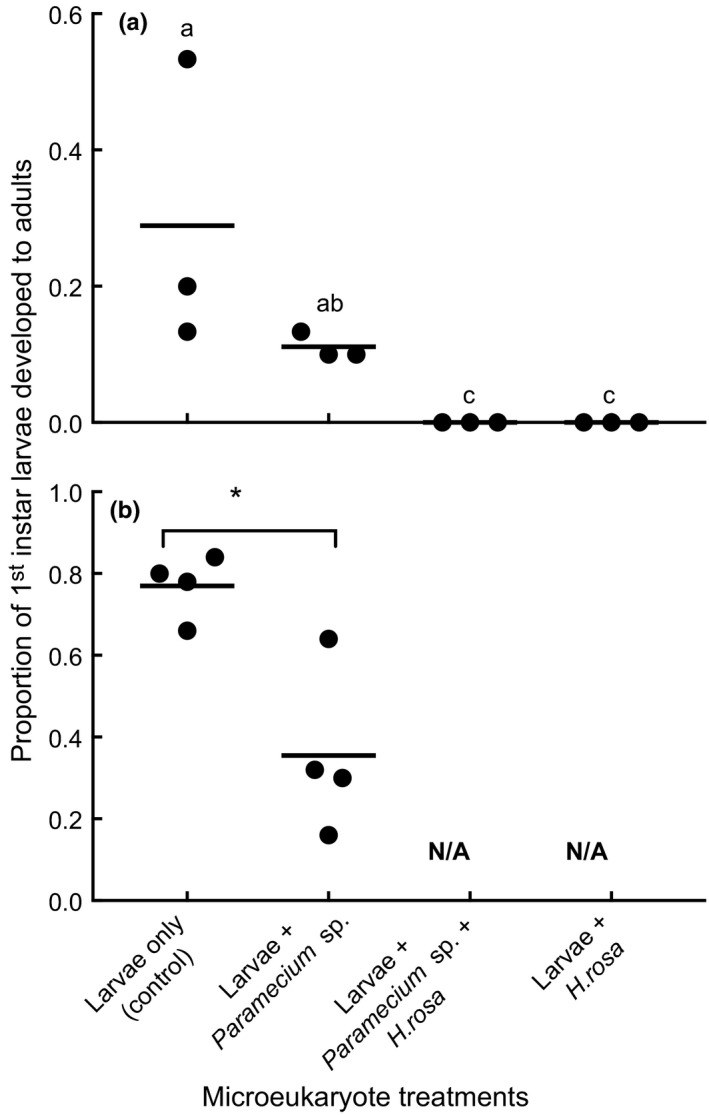
Scattered plot (mean, *n* = 3) of proportion of first instar larvae to adult survivorship in two microeukaryote species treatments (*Paramecium* sp. and *Habrotrocha rosa*), and untreated control in the first experiment (a). No adults developed from larvae in the *H. rosa* and a combination of *H. rosa* and *Paramecium* sp. treatments. Mean (*n* = 4) proportion of total adults developed from first instars of *Cx. nigripalpus* larvae exposed to *Paramecium* sp. and untreated control in the second experiment (b). No *H. rosa* and a combination of *H. rosa* and *Paramecium* sp. treatments were used in the second experiment. The means are indicated by horizontal line. Significantly different means are shown in lower case letters or asterisk

The mean proportions of adults developed from first instar larvae of *Cx. nigripalpus* were significantly different among the treatments in the first experiments (Figure 1a; Kruskal–Wallis Test: χ^2^ = 9, *p* = .028, *df* = 3). Addition of 247 *Paramecium* sp. (±23 SE) per ml, 84 *H. rosa* (±6.5) per ml, or 172 individuals of the combination of the two species (±6) per ml into mosquito larval food base significantly reduced or prevented successful mosquito development compared to untreated control treatments. A total of 36 adults (13 females and 23 males) emerged from this experiment, 72% of which were from untreated control treatment. The remaining 28% emerged from *Paramecium* sp. only treatment, and no adults emerged from *H. rosa* or a combination of the two species treatments until 14 days.

In the second experiment, there was a similar depression of mosquito production from containers that received *Paramecium* treatments (Figure 1b, Table 1). Significantly lower proportions of first instar larvae developed to adulthood in containers with 29.6 *Paramecium* sp. (±1.95 SE) per ml than in untreated control containers (Figure 1b, Table 1). On average, nearly 72% (±5 SE) of larvae in the untreated control developed to adulthood, while only 34% (±9 SE) of the first instar larvae developed into adults in containers with *Paramecium* sp. A total of 112 (76 from untreated control and 36 from *Paramecium* sp. treatment) females and 100 males (68 from untreated control and 36 from protozoa) developed to adulthood in the second experiment.

**Table 1:** Results of MANOVA followed by univariate analyzes of the effects of microeukaryote treatments on adult weight and larvae to adult development time of *Culex nigripalpus* in experiments 1 and 2. Degrees of freedom reported for univariate analysis are for treatments and errors and different from that of MANOVA, respectively. Significantly different effects were indicated by bold *p*‐values

	*F*	DFNum	DFDen	*p*
Experiment 2				
MANOVA: *F*‐test	1411.32	6	1	**.0204**
Univariate analysis				
Variable				
Average individual female weight	16.44	1	6	**.0067**
Average female emergence time	2.14	1	6	.1933
Total female weight	12.097	1	6	**.0132**
Average individual male weight	16.23	1	6	**.0069**
Average male emergence time	0.095	1	6	.7686
Average number of adults	13.13	1	6	**.0111**
Experiment 3				
MANOVA Roy's Max Root	114.19	6	8	**<.0001**
Univariate analysis				
Variable				
Average individual female weight	38.696	3	10	**<.0001**
Average female emergence time	11.68	3	10	**.0013**
Total female mass	3.54	3	10	.0558
Average individual male weight	41.67	3	10	**<.0001**
Average male emergence time	9.96	3	10	**.0024**
Average number of adults	1.34	3	10	.3191

**Figure 2:**
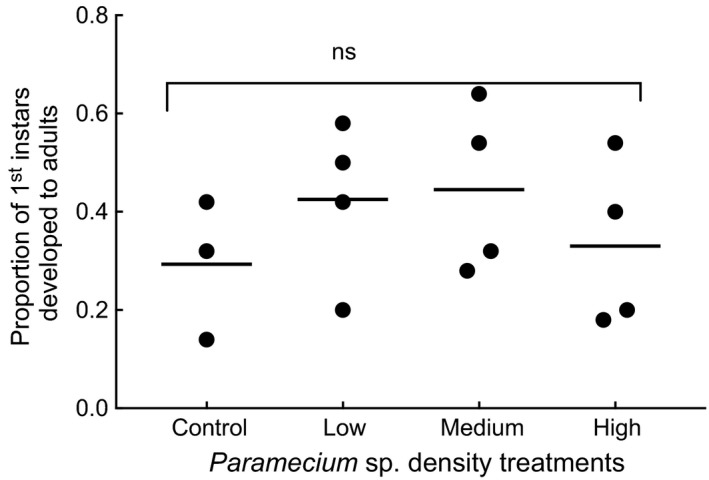
Scattered plot (mean, *n* = 4) of proportion of adults developed from first instars of *Culex nigripalpus* larvae exposed to three densities (high = 40 ± 1.4 protists/ml, medium = 16.7 ± 3, and low = 1.7 ± 1) of *Paramecium* sp. treatments, and untreated control. Means were not significantly different (ns)

In the third experiment, the mean adult proportions emerged from the different *Paramecium* sp. density treatments were not significantly different (Figure 2, Table 1). A total of 102 female adults (18, 34, 34, and 16 females) and 177 male adults (24, 51, 54, and 48 males) developed from 1st instar larvae to adulthood raised in containers that received 0 (untreated control), low (1.7 *Paramecium* sp. per ml), medium (17), and high (40) *Paramecium* density treatments, respectively, by day 14 from all treatments. Five other adults that include one individual from medium treatment, two from each of high and untreated control emerged after 14 days, and were not included in the analyzes.

#### 3.1.2 Emergence time

**Figure 3:**
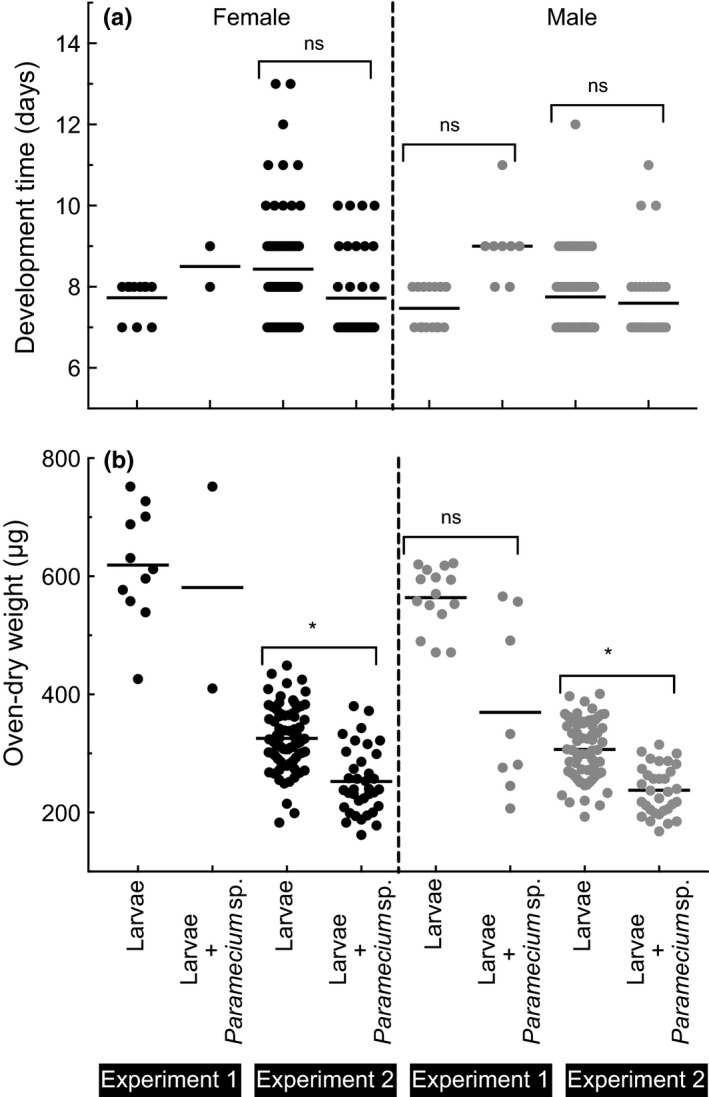
Scatter plot of adult development time (upper panel) and oven‐dried adult weight (lower panel) of *Culex nigripalpus* developed from the first instar larvae exposed to microeukaryotes in the first and second experiments. Individual measurements from females (solid circle) and males (gray circle) are shown in left and right panels, respectively. * = significantly different (*p* < 0.05); ns = not significantly different (*p* > 0.05)

**Figure 4:**
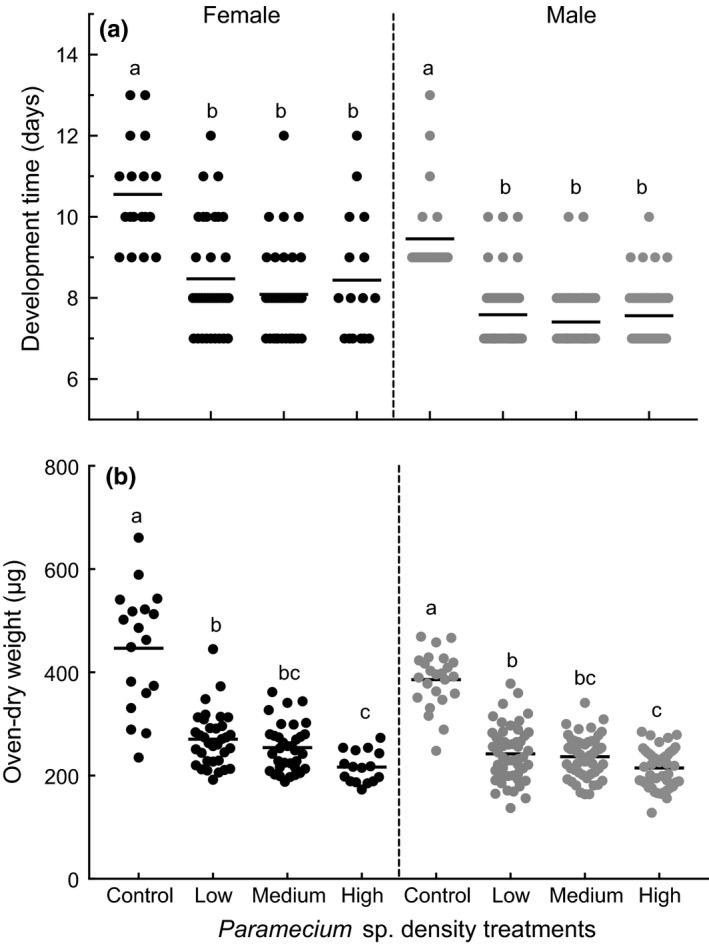
Scatter plot of adult development time (a) and oven‐dried adult weight (b) of *Culex nigripalpus* females (soild circles, left panel) and males (gray circle, right panel) developed from containers that received four densities of *Paramecium* sp. during the third experiment

Differences in larval development time to female emergence were not assessed among the treatments in the first experiment because only two female adults emerged from containers that received *Paramecium* sp. and none from rotifer and the combination of the two species treatments (Figure 3a). Larval development time to female adulthood was slightly reduced in containers that received *Paramecium* sp. treatment compared with development time of female adults in untreated control in the second experiment, but the difference was not statistically significant (Figure 3a, Table 1). On average, it took 7.6 (±0.5 SE) and 8.4 (±0.3) days for the first instar larvae to develop to female adults in containers with and without *Paramecium* treatments, respectively. Approximately equal time (7.9 days) was taken for larvae to develop to male adults in containers with and without *Paramecium*. Larval development time to female adulthood was significantly reduced (Figure 4a, Table 1) in containers that received three densities of *Paramecium* sp. treatments compared with development time in untreated control treatments in the third experiments. Males generally developed faster than females.

#### 3.1.3 Average individual adult weight

The ciliate protist treatments significantly reduced the individual biomass of adult mosquitoes in all experiments (Figures 3 and 4). Female mosquitoes emerging from treatments with microeukaryote additions weighed less than mosquitoes reared without the addition of the two microbial treatments in the first experiment, although the difference could not be statistically tested due to the low number of adults emerged (Figure 3a). The mean dry weight of females was 581 μg (±171 SE) and 637 μg (±26) in *Paramecium* sp. treatments and the untreated control, respectively.

Females that developed from containers that received *Paramecium* sp. treatments weighed significantly less than females developed from treatments without *Paramecium* sp. in the second experiment (Figure 3b, Table 1). The mean weight of females emerged from *Paramecium* treatments was 232 μg (±22 SE) compared with 324 μg (±7) in untreated control. The effect of *Paramecium* sp. on males was also significantly different. The mean weight of males was 229.8 µg (±15) and 313.5 µg (±13) from treatments with and without *Paramecium* treatments, respectively.

The different densities of *Paramecium* sp. treatment also significantly affected the biomass of the both adult females and males (Figure 4b, Table 1) in the third experiment. Mosquitoes emerging from untreated containers had the greatest individual adult weight, followed by the low and medium treatments. The high *Paramecium* sp. density treatment resulted in the lowest average adult weight.

#### 3.1.4 Total adult biomass

In the first and second experiments, female mosquito production was significantly depressed by the additions of microeukaryotes, but this was not the case in the third experiment (Figure S2, Table 1). In the third experiment, the smaller average size of females in the *Paramecium* treatments was offset by slightly higher numbers of females emerging, resulting in overall biomass levels that were similar to the control treatment.

### 3.2 Effects of mosquito larvae on microeukaryote population dynamics

**Figure 5:**
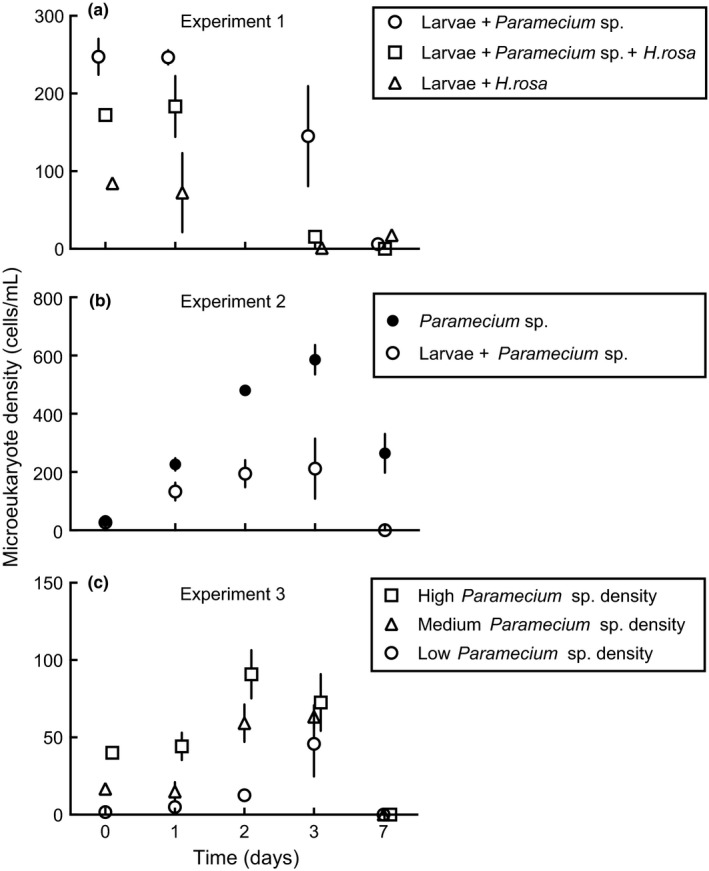
Mean ± SEM (n = 3) of *Paramecium* sp., *Habrotrocha rosa* Donner, and a combination of *H. rosa* and *Paramecium* sp. population dynamics in the water column following the introduction of *Culex nigripalpus* first instar larvae in the first experiment (a); *Paramecium* sp. in the water column with (solid circle) and without (open circle) *Cx. nigripalpus* larvae in the second experiment (b); and three *Paramecium* sp. density treatments in the presence of *Cx. nigripalpus* larvae in the third experiment (c). The *x*‐axis was offset ±0.1 days for better illustration where treatment data points overlapped

In the first experiment, microbial population abundance in the three treatments was significantly different (Figure 5a, Table 2). *H. rosa* and *Paramecium* populations declined across sampling dates and were nearly undetectable in the water column a week after the introduction of these microbes. In the second experiment, the abundance of *Paramecium* sp. also differed significantly among treatments (Figure 5b, Table 2). *Paramecium* sp. abundance increased linearly until 72 hr and then declined to a level nearly undetectable level after a week in treatments that contained mosquito larvae. A similar pattern was observed in the third experiment (Figure 5c, Table 2), but a lag in *Paramecium* population growth during this experiment was observed compared with the second experiment because initial bacterial densities were much lower in freshly prepared media versus media that had been incubated for 4–10 days.

**Table 2:** Results of mixed model analyzes of the effects of treatments on abundance of small (0.2–2.0 μm ESD) and large (2–60 μm ESD) particles in water column subjected to microeukaryotes and mosquitoes in the three experiments across different dates. Significantly different effects were indicated by bold *p*‐values

Parameters	Experiments	Source	*F*	DFNum	DFDen	*p*
Small particles	1	Treatment	127.5	3	8	**<.0001**
		Date	90.5	3	6	**<.0001**
		Treatment × Date	9.4	9	6.9	**.0040**
	2	Treatment	56.3	2	9	**<.0001**
		Date	110.8	3	7	**<.0001**
		Treatment × Date	27.8	6	8.1	**.0040**
	3	Treatment	1.1	3	11	.3926
		Date	52.4	3	10	**<.0001**
		Treatment × Date	6.8	6	11.7	**.0027**
Large particles	1	Treatment	51.0	3	8	**<.0001**
		Date	46.1	3	6	**.0002**
		Treatment × Date	6.9	9	9	**.0283**
	2	Treatment	15.4	2	9	**.0012**
		Date	25.6	4	6	**.0007**
		Treatment × Date	6.2	8	7	**.0132**
	3	Treatment	9.56	3	11	**.0021**
		Date	172.6	2	10	**<.0001**
		Treatment × Date	14.2	6	11.7	**<.0001**
Microeukaryotes	1	Treatment	28.8	2	6	**.0008**
		Date	128.2	3	4	**.0002**
		Treatment × Date	10.3	6	4.4	**.0160**
	2	Treatment	625.1	1	6	**<.0001**
		Date	1580.1	4	3	**<.0001**
		Treatment × Date	648.8	4	3	<**.0001**
	3	Treatment	47.1	2	9	**<.0001**
		Date	34.3	3	7	**.0009**
		Treatment × Date	0.9	6	8.1	.0509

### 3.3 Effects of microeukaryotes on abundance of organic particulates and bacteria

**Figure 6:**
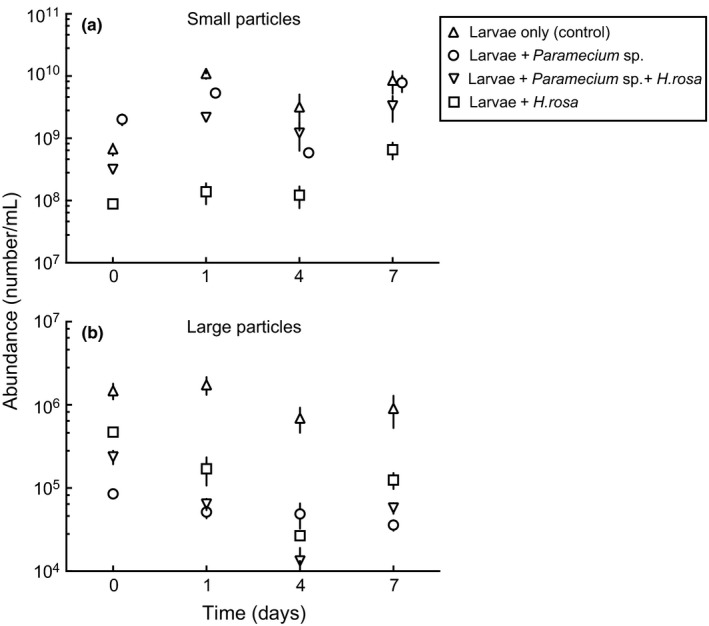
Mean ± SEM (*n* = 3) number of small (0.2–2.0 μm ESD, a) and large (2.0–60 μm ESD, b) organic particle dynamics in the hay media with and without *Paramecium* sp., with a combination of *Paramecium* sp. and *Habrotrocha rosa*, and with only *H. rosa* treatments

The abundance of small organic particles (size: 0.2–2 μm ESD), which also includes many bacteria varied significantly among the treatments in the first experiment (Figure 6a, Table 2). The abundance of this particle size group was significantly lower in the *H. rosa* treatments (Figures 6a and S3). The abundance of large particles (2–60 μm ESD) was significantly reduced in both microeukaryote addition treatments, with greater concentration of particles found in the untreated controls (Figure 6b, Table 2). Although some differences were detected, abundance of both size ranges remained generally stable across sampling dates during this experiment.

**Figure 7:**
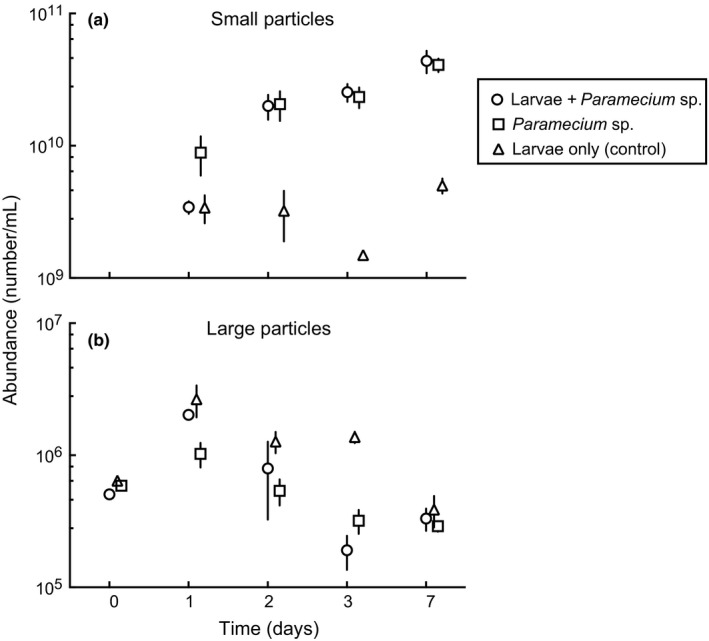
Mean ± SEM (*n* = 4) total small (a) and large (b) organic particle (cell) dynamics in the hay media with and without *Paramecium*, and in hay media with *Paramecium* only treatments

In the second experiment, the abundance of small particles (0.2–2 μm) was significantly different among treatments with greater abundance of this particle group found in water with *Paramecium* sp. (Figure 7a, Table 2). Mosquito larvae in treatments with *Paramecium* sp. did not affect the abundance of small particles, while this size class was significantly reduced in treatments without *Paramecium* sp. In contrast, the abundance of large particles (2–60 μm) was significantly greater (Figure 7b, Table 2) in containers without *Paramecium* sp. treatment. Differences in large particle counts were not apparent among the three treatments after a week. Microscopic examination of water column samples revealed that a flagellate protist sp. (size: 3–5 μm ESD) dominated the large size particle group (Figures S5 and S6). The *Paramecium* sp. apparently preferentially consumed these flagellates in the presence or absence of mosquito larvae (Figure S5). Whereas there was no difference in small particle abundance between treatments with and without ciliate protist on day 1, this particle group was significantly reduced by approximately 84%, 94%, and 89% in days 2, 3, and 7, respectively, in treatments that contained mosquito larvae but with no *Paramecium* sp. (Figures 7a, S6).

**Figure 8:**
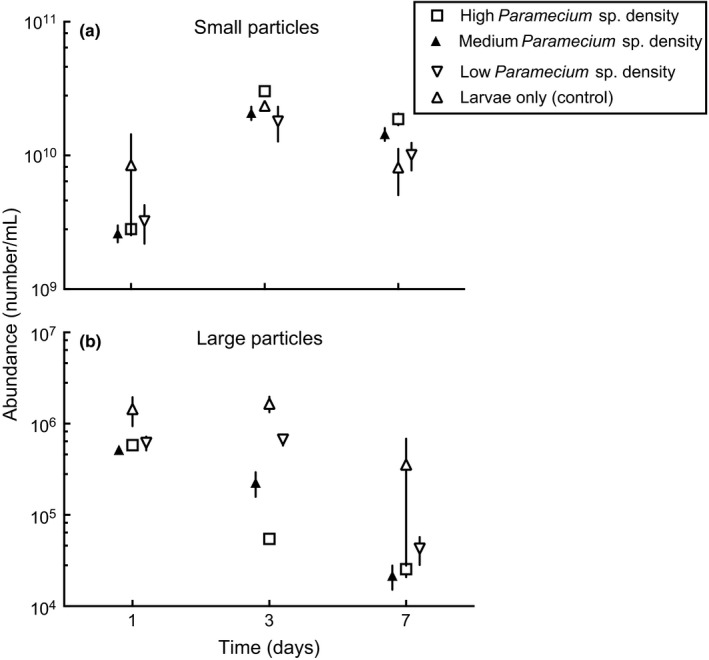
Mean ± SEM (*n* = 4) total small (a) and large (b) particle (cell) dynamics in the hay media containing *Culex nigripalpus* larvae and four different *Paramecium* sp. density treatments

In the third experiment, the effect of treatments on smaller particles was not statistically significant among the various *Paramecium* sp. density treatments (Figure 8a, Table 2). However, the abundance of the large particle group was consistently and significantly depressed in containers with ciliate protists in a density‐dependent manner (Figure 8b, Table 2). Untreated controls (i.e., only mosquito larvae introductions) had the greatest abundance of large particle group.

### 3.4 Interactions of mosquito parameters with microeukaryotes and sestonic particles

Results of a structural equation model revealed that the proportion of first instar larvae developing to the adult stage had a differential effect on the two particle size ranges, although the maximum likelihood estimate was not significant (Table S1). The presence of mosquitoes had a significant negative impact on microeukaryote (ciliate protist and/rotifer) populations. Interestingly, a high number of emerged female mosquitoes had a positive effect on the abundance of large particles. High average individual mosquito weights significantly reduced the abundance of small particles and microeukaryote populations. In contrast, microeukaryotes significantly reduced large particle abundance, and appeared to have a positive effect on the small particles.

## 4 Discussion

### 4.1 Microeukaryote effects on mosquito larval development and adult biomass

Our study revealed that addition of ciliates and rotifers altered larval mosquito resources and negatively affected larval development and biomass of *Cx. nigripalpus* mosquitoes in a laboratory environment. These results are contradictory to previous assumptions that an addition of free‐living microeukaryotes would enhance mosquito development by serving as food resources for larvae. These two eukaryotic components of the planktonic communities in container systems are considered to be important grazers of bacteria, providing a direct link in transferring bacterial biomass to a larger aquatic invertebrates including mosquito larvae (Porter et al., 1979; Sanders & Wickman, 1993). Our experiments indicated that at least in the cases of the protists and rotifer species used here, very little of that bacterial biomass transfer occurred and most likely these microeukaryotes competed with mosquito larvae for small size class microbial biomass.

Although the negative effects of these microbes on mosquito development probably resulted from competition with early instar mosquito larvae for smaller organic particulates, we cannot rule out other factors associated with the ciliates and rotifers used here that might have inhibited larval development. A notable example of inhibition by a free‐living commensal protist has been observed for *Lambornella clarki* Corliss and Coats (Cilophora: Tetrahymendiae) interactions with larvae of the western tree hole mosquito, *Aedes sirrensis* Ludlow. Under intense larval predation pressure, *L. clarki* transformed into a parasitic form, causing significant *Ae. sierrensis* larval mortality (Washburn, Gross, Mercer, & Anderson, 1988). *Culex* mosquitoes are generally considered filter feeders of microorganisms in the water column and particularly feed on bacteria, differing from *Aedes* mosquitoes in their feeding habit in that they graze less on the substrates and more on planktonic protists (Merritt, Dadd, & Walker, 1992). We did not detect parasitic protist forms during our study, but we also did not measure larval mortality during the course of the experiments. We have also not excluded other possibilities such as symbiont‐mediated parasitism/pathogenesis that has been well documented elsewhere (Gortz & Brigge, 1998; Price et al., 1986). The fact that a rotifer also inhibited larval development in our study argues for a more general competitive interaction between microeukaryotes and mosquito larvae, and also suggests that at least in the case of early instars, *Culex* larvae are unable to utilize larger microeukaryotes as food resources. In a pitcher plant system, protists and rotifers have also been shown to reduce the larval development of *Wyeomyia smithii* Coq., presumably through competitive interactions (Kneitel, 2007).

It is likely that effects of inter‐phyletic competition described here and elsewhere (Lounibos, 2007) between the microeukaryotes and *Cx. nigripalpus* larvae contributed to differences in adult emergence rates as well as the size of the adults. Larger wing size (predictor for adult weight) and an extended larval to adult development period due to the effects of intraspecific larval competition was previously reported for *Aedes* mosquitoes (Alto, Lounibos, Mores, & Reiskind, 2008). Any surviving larvae, released from the effects of intraspecific competition or other causes of larval mortality, tend to develop to adults more slowly, resulting in higher body weight compared to mosquito larvae developing under other, more favorable conditions. We did not observe this in our study because lower survival rates were associated with smaller adults and shorter emergence times. This suggests that mortality of conspecific larvae did not release remaining larvae from competition and that the survivors continued to compete with the added microeukaryotes for resources, while further investigation is required to determine the exact causes of the reduced adult size in this study in microeukaryote treatments compared to the untreated controls, it points out another possible factor in larval habitats that can influence production and disease transmission capacity of mosquito vectors.

The lack of differences in mosquito production among different density treatments of protists in the third experiment, compared to the first two, was likely due to the effects of a delay in bacteria colonization of the medium at the time of larval and *Paramecium* sp. addition. Microbial resources (bacteria and flagellates) were relatively scarce in the hay medium on day 0, and this may have affected growth of mosquito larvae in all the treatments as well as growth of the *Paramecium* sp. In addition, *Cx. nigripalpus* larvae used in this study were derived from field populations collected during three different time periods (October, February and March), and some of the growth performance differences observed among the three experiments might be attributable to genetic differences in populations of mosquitoes used in these experiments (Nayar, Knight, & Munstermann, 2002).

### 4.2 Effects on lower trophic microorganisms and organic particulates

The populations of the two microeukaryotes (rotifer and protists treatments) declined over time due to a combination of factors, including ingestion by mosquito larvae, the reduction of the food (smaller microbial resources) in the water column, and unknown factors associated with the presence of mosquito larvae. Ciliate population growth ceased by day three (Figure 5b,c, Table S1) both in the presence or absence of mosquito larvae, suggesting that their decline was more likely due to the depletion of food resources (bacteria) than consumption by *Cx. nigripalpus* larvae.

A high throughput particle analysis of both small (0.2–2 μm ESD) and large (2–60 μm) organic particles and similar sized microbes revealed mosquito larvae differentially affected these lower trophic microorganisms (particles) in the presence or absence of microeukaryotes. These results collectively indicate that early instar *Culex* and bacterivorous microeukaryotes compete intensely for small particle resources and that *Culex* larvae are not necessarily able to utilize all microbial resources equally. This was particularly true when the rotifer, *H. rosa*, was added to larval containers. In our study, *H. rosa* reduced the growth of bacteria and other bacterial sized cells and prevented larval mosquitoes from developing into adulthood (Figure 1). Rotifers are known to graze heavily on bacteria (Snell & Hicks, 2011) and are also considered predators of ciliate protists and other microbial consortia (Arndt, 1993). Rotifers depleted bacterial sized resources even more than ciliate protists, and thus were likely stronger competitors with young mosquito larvae (Figures 6 and S4). *H. rosa* is considered an obligate occupant of pitcher plants and was shown to consume more bacteria than *Wyeomyia smithii* mosquito larvae (Hoekman, 2011; Kneitel, 2007; Petersen, Hanley, Walsh, Hunt, & Duffield, 1997).

Flagellate (3–5 μm ESD) abundances significantly increased in treatments that only contained *Cx. nigripalpus* larvae compared to containers that had both *Cx. nigripalpus* larvae and *Paramecium* sp. (Figures S5 and S6), possibly because these flagellates are not readily ingestible or digestible by early instars. These groups decreased in the presence of ciliate protists (Figures S5 and S6, Table S1) regardless of the presence of mosquito larvae, suggesting that ciliates apparently ingest/digest particles in the 3–5 μm size range more efficiently than 1st instar mosquito larvae despite their small size relative to early instar mosquito larvae. Small particles, including bacteria, however, increased in the presence of ciliate protists (Figure 7a and Figure S5, Table S1) regardless of the presence of mosquito larvae. Protists and mosquito larval grazing did not influence overall bacterial abundance in pitcher plant systems (Hoekman, 2011), whereas in this study mosquitoes reduced bacteria cells and other small particles <2 μm ESD from the water column when *Paramecium* sp. was absent. *Culex* larvae preferentially removed bacteria and bacteria–sized cells (Figures S5 and S6), suggesting the importance of bacteria for successful larval development. However, because the bacterial community composition is unlikely to be static during the course of the experiment or with treatments, further investigation is required to determine whether specific groups of bacterial taxa are required for larval development of *Culex* species.

Tree hole inhabiting mosquitoes such as *Aedes triseriatus* Say are known to be key top predators and can have a profound negative effect on both flagellate and ciliate abundance and composition in tree hole systems (Kaufman, Goodfriend, Kohler‐Garrigan, Walker, & Klug, 2002). In our study, *Culex* larvae appear to be primarily feeding on bacterial communities as opposed to larger and potentially more nutritious microbes such as flagellates or ciliates during early instar stages. Overall, microbial abundance in the hay culture of our study is relatively high compared to most previously studied larval mosquito habitats (e.g., Kaufman et al., 2002). However, these levels can be typical of the microbial abundance found in polluted *Culex* habitats such as treatment wetlands (Peck & Walton, 2008), and the feeding strategies of larval *Culex* and *Aedes* are likely to differ.

## 5 Conclusions

Our study provided evidence that increased abundance and diversity of microeukaryotes in the larval habitat might significantly reduce abundance as well as individual female biomass of adult *Culex* mosquitoes. Both of these parameters are known to affect disease transmission and population dynamics of mosquitoes. Fewer and smaller *Cx. nigripalpus* female mosquitoes developed from 1st instar larvae exposed to rotifer and ciliate protists; a consequence of altered larval resources (bacteria and other microbes). Higher mosquito production was associated with a lower abundance of small particles, including bacteria, in the absence of the ciliate protists, indicating that *Cx. nigripalpus* larvae are relying heavily upon bacteria for food particularly during early instar stages. This may explain part of the observed preference for some species of *Culex* mosquitoes, including *Cx. nigripalpus*, to utilize highly enriched larval habitats compared to other mosquito groups. Future studies will investigate the combination of these microbial communities and mosquito microbial larvicides on mosquito production from natural habitats.

## Conflict of Interest

None declared.
