# Supplementary material for: Aquatic microfauna alter larval food resources and affect development and biomass of West Nile and Saint Louis encephalitis vector Culex nigripalpus (Diptera: Culicidae)
Source: Ecol Evol. 2017 Apr 9;7(10):3507–19. doi: 10.1002/ece3.2947 (PMC5433994; doi:10.1002/ece3.2947)

**Supporting Information**

**Aquatic microfauna alter microbial larval resources and affect larval development and biomass of West Nile and Saint Louis encephalitis vector *Culex nigripalpus* (Diptera: Culicidae)**

D. Duguma*^1^ M. G. Kaufman^2^, A. B. Simas Domingos^1^

The following Supporting Information is available for this article online.

Table S1. The maximum likelihood standardized path estimates of the SEM model using PROC CALIS involving mosquito food web components: small (0.2-2.0 µm ESD, S) and large (2-60 µm ESD, L) sestonic particles, microeukaryotes (a bdelloid rotifer and a ciliate protist, M), proportion of first instar larvae developed to adults (T), female (T'), and average adult weight. Significant estimate values are indicated in bold (df=19).

| Standardized Results for PATH List | | | | | | |
| --- | --- | --- | --- | --- | --- | --- |
| **Path** | | | **Parameter** | **Estimate** | **Standard Error** | **t value** |
| Small particles | <=== | Proportion | T_S | -0.1843 | 0.1891 | -0.9748 |
| Large particles | <=== | Proportion | T_L | 0.0968 | 0.1455 | 0.6652 |
| Microeukaryotes | <=== | Proportion | T_M | -**0.3866** | 0.1607 | -2.4054 |
| Small particles | <=== | Microeukaryotes | M_S | 0.2379 | 0.1872 | 1.2708 |
| Large particles | <=== | Microeukaryotes | M_L | -**0.6603** | 0.1161 | -5.6883 |
|  |  |  |  |  |  |  |
| Small particles | <=== | Female | T'_S | -0.1603 | 0.1862 | -0.8609 |
| Large particles | <=== | Female | T'_L | 0.2359 | 0.1356 | 1.7391 |
| Microeukaryotes | <=== | Female | T'_M | -0.3337 | 0.1679 | -1.9867 |
| Small particles | <=== | Microeukaryotes | M_S | 0.2557 | 0.1828 | 1.3986 |
| Large particles | <=== | Microeukaryotes | M_L | **-0.61899** | 0.1139 | -5.4354 |
|  |  |  |  |  |  |  |
| Small particles | <=== | Average weight | T''_S | **-0.4107** | 0.1596 | -2.5729 |
| Large particles | <=== | Average weight | T''_L | -0.0464 | 0.1460 | -0.3175 |
| Microeukaryotes | <=== | Average weight | T''_M | **-0.3811** | 0.1556 | -2.4498 |
| Small particles | <=== | Microeukaryotes | M_S | 0.1527 | 0.1768 | 0.8637 |
| Large particles | <=== | Microeukaryotes | M_L | **-0.7154** | 0.1094 | -6.5376 |

**Fig. S1**. *Paramecium* sp. (left) and *Habrotrocha* *rosa* Donner (right) used in the experiemtns


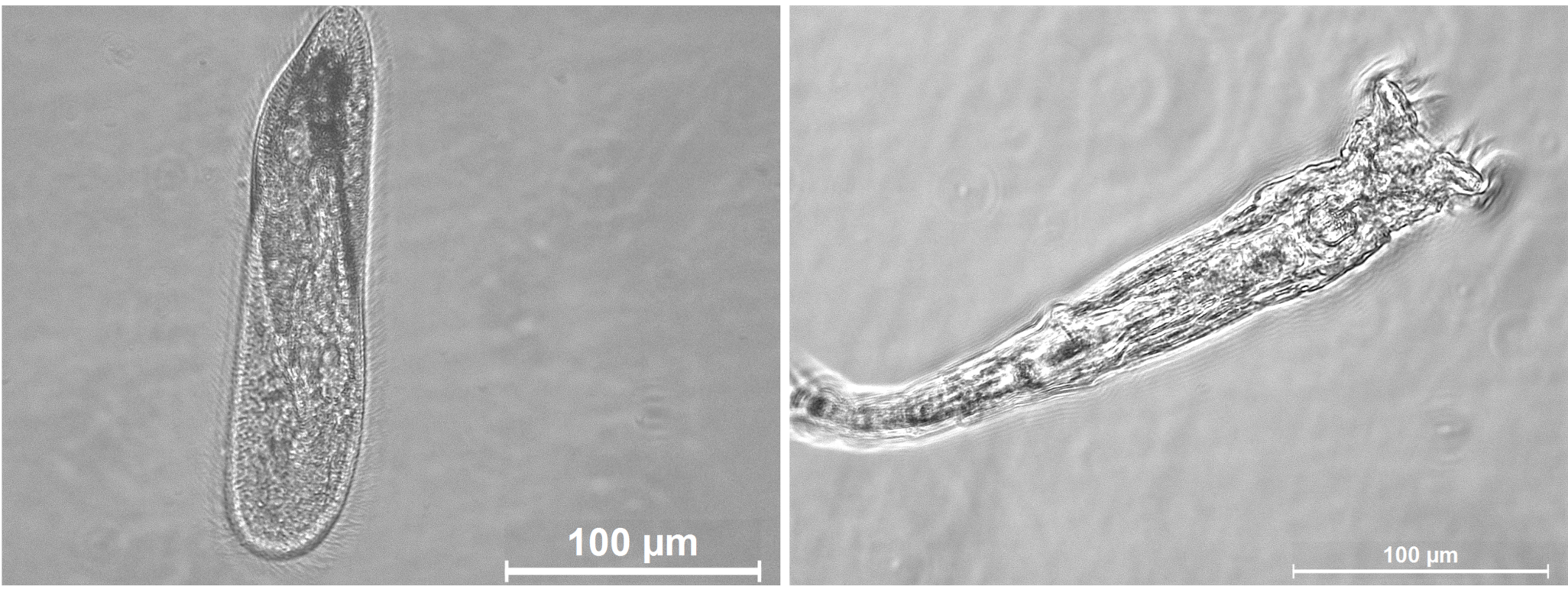


Fig. S2. Total mosquito production. Scattered plot of total biomass of female adult (solid circles) and male (gray circles) mosquitoes developed from larvae exposed to microeukaryotes in the first (A), second (B), and third experiments (C). “ *” = Means were significantly different (p <0.05), ns= not significant.

**Fig. S3.** Organic particle dynamics in water column. Particle (cell) dynamics range between 0.2–2 µm (upper panel), and 2–10 µm (lower panel). Upper panel: particle (cell) dynamic size ranging between 0.2–2µm in the water following introduction of larvae of mosquitoes with ciliate protists (orange line), rotifers and ciliate protozoa (gray line), with rotifers only (yellow line) and untreated control (i.e., without the addition of the microbes, blue line). Lower panel particle (cell) dynamics size ranging between 2–5 µm in the water following introduction of *Culex* larvae (blue line) and ciliate protists (green line), and both mosquito larvae and ciliate protozoa in water (lower panel).

**
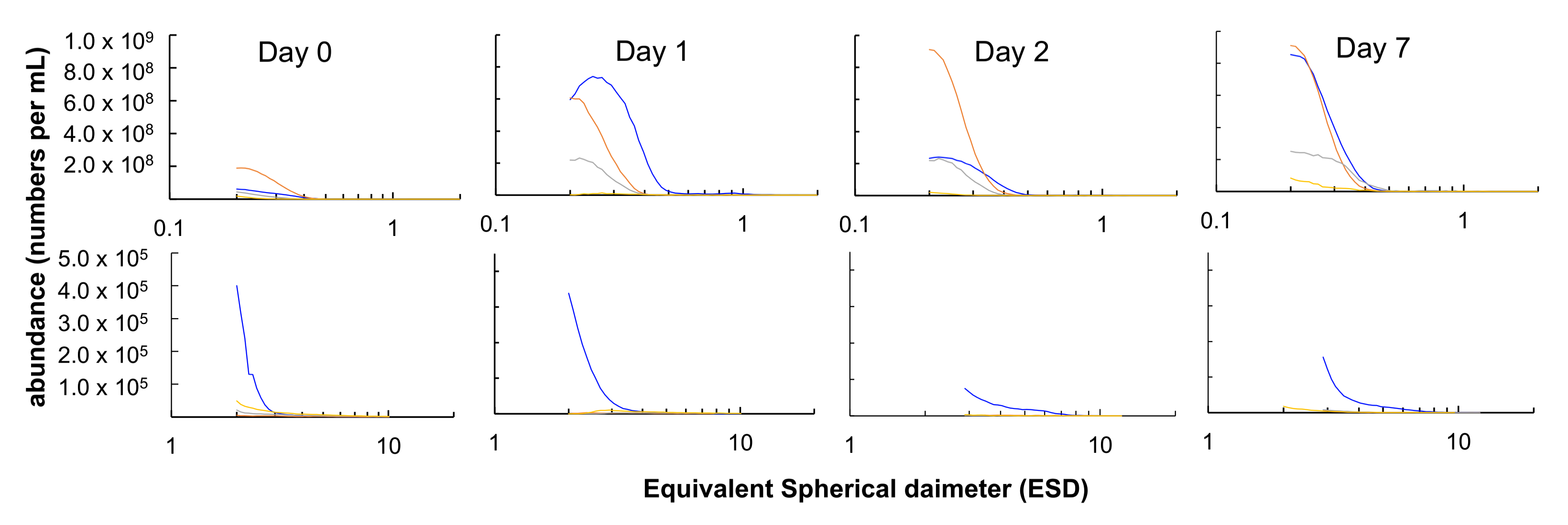
**

**Fig. S4.** Organic nanoparticle dynamics in water column. Upper panel: particle (cell) dynamic size ranging between 0.2–2µm ESD in the water column containing *Culex* larvae without (blue line) and with *Paramecium* sp. (orange line), and in water column only containing *Paramecium* sp. (gray line); Lower panel particle (cell) dynamics size ranging between 2–60 µm in the water following introduction of *Culex* larvae. *Small particles in the water column were not measured on day 0.


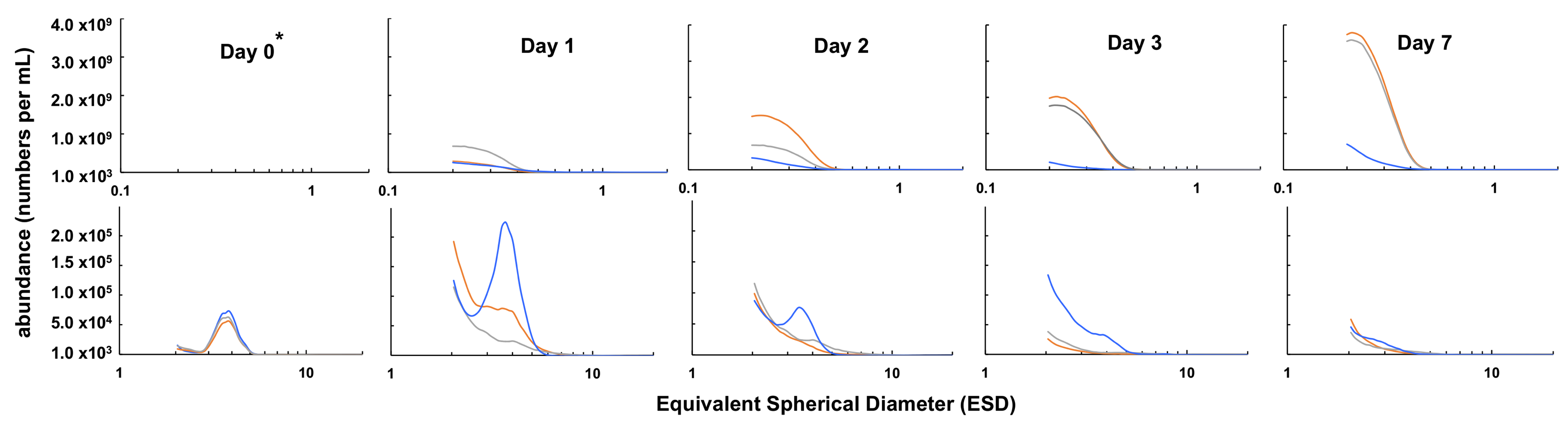


**Fig. S5**. *Bacteria* and other microbial cells in water column. Microbial cells in water with (A) and without (B) *Paramecium* sp. treatment 24 h after introduction *Culex* *nigripalpus* first instar larvae (40X). Reduced abundance of flagellates (size 3–5µm ESD) and increased bacteria in treatments with *Paramecium* sp. were observed (Panel A), whereas the number of flagellates in treatments without *Paramecium* sp*.* increased while significantly depressing *Bacteria* populations. Treatment that contained only *Paramecium* sp. treatment without mosquito larvae was similar to Panel A, and is not shown here.


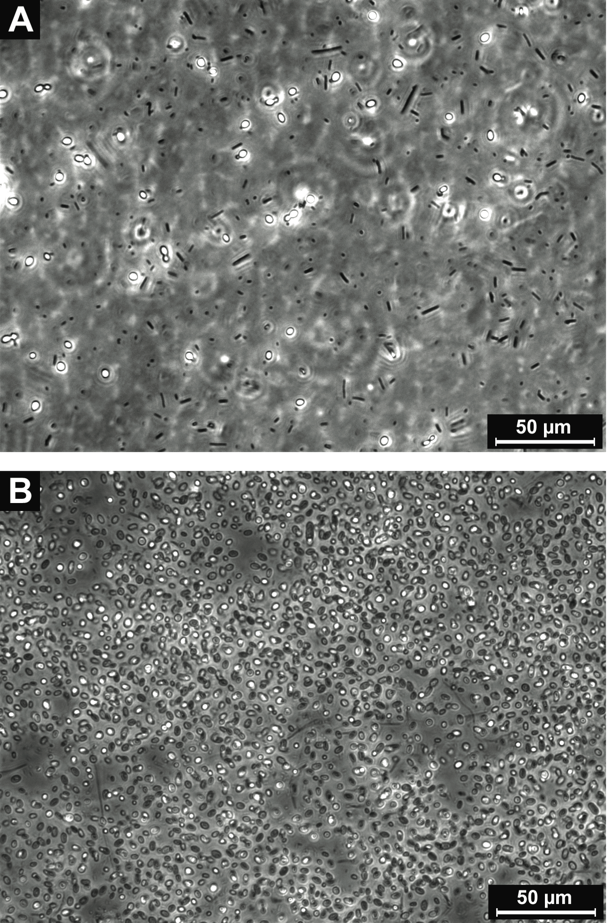


**Fig. S6.** *Bacteria* and other microbial cells in water column. *Bacteria* in water with (A & C) and without (B) *Paramecium* sp. treatment 72 h after introduction *Culex* *nigripalpus* first instar larvae in A & B. Magnification was made at 40X.


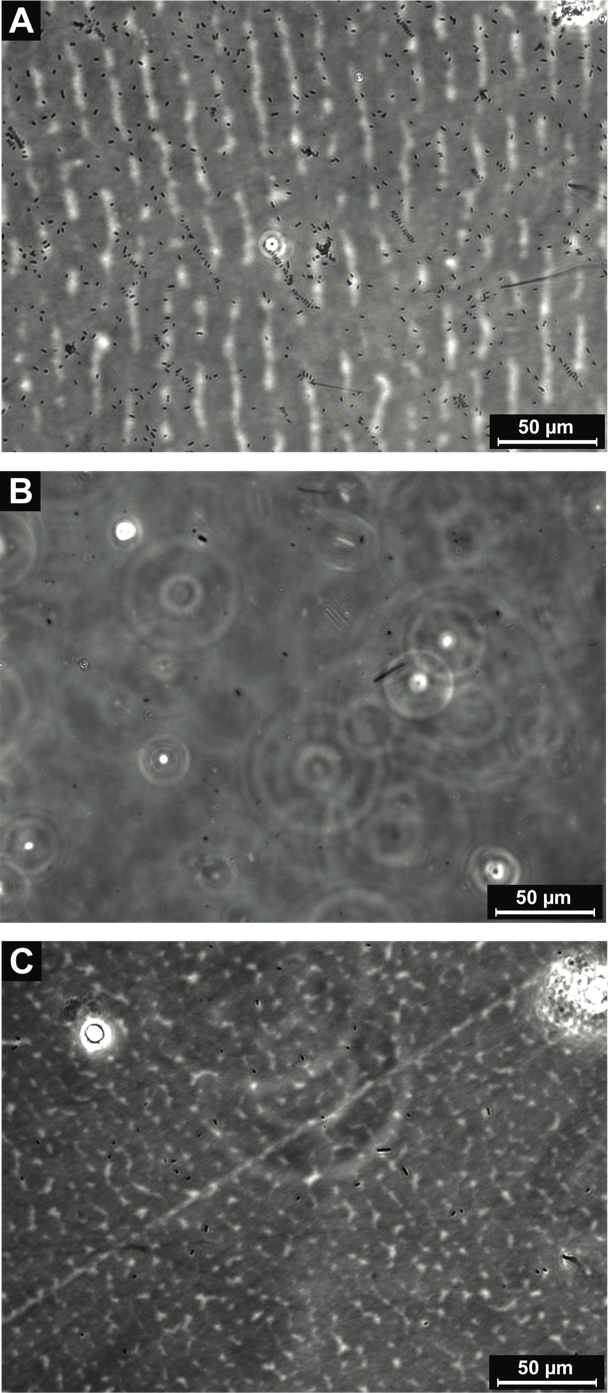


**Fig. S7.** Flagellates size range between 3–5 µm ESD ingested (indicated by arrow) by *Paramecium* sp.


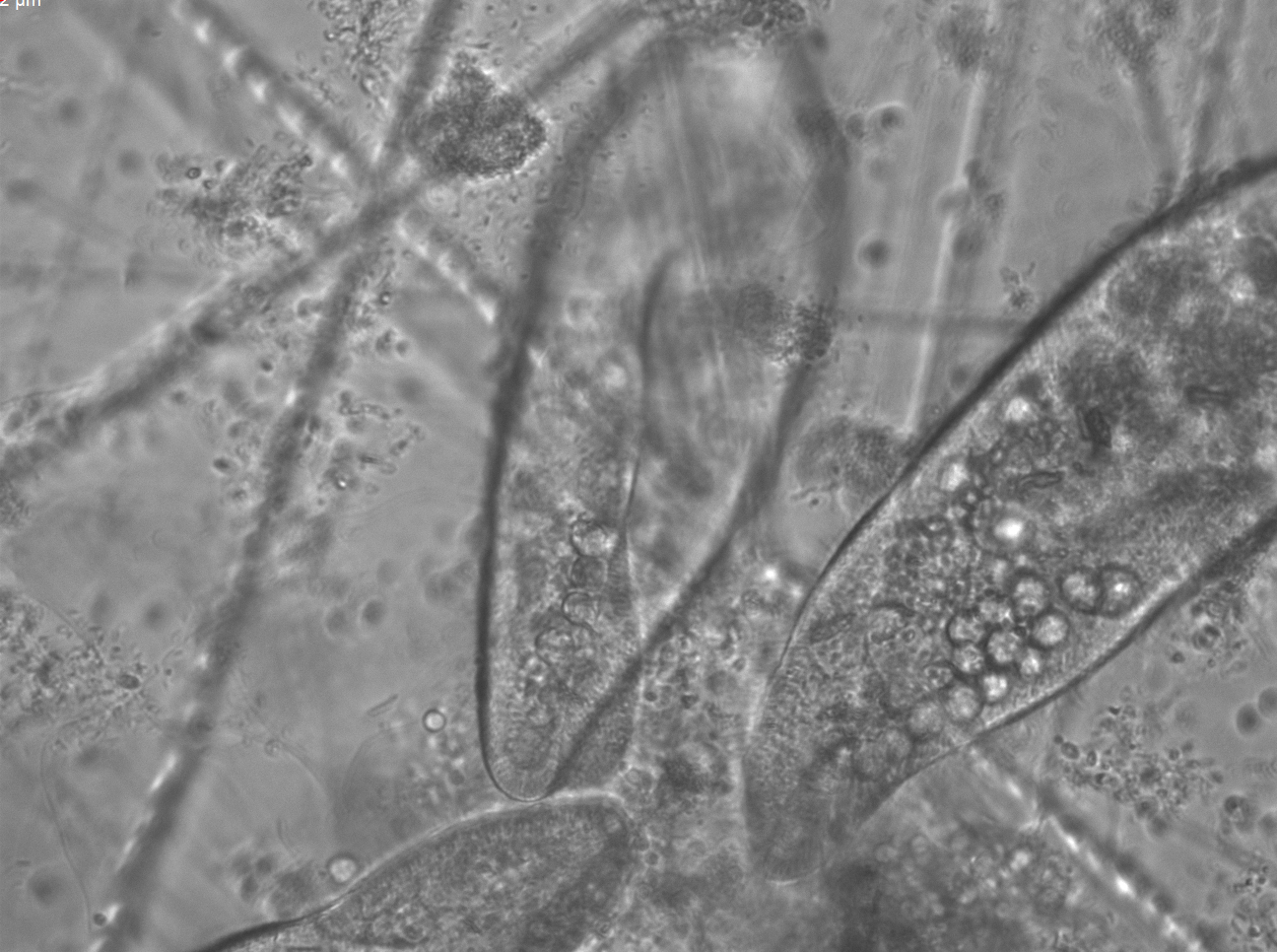

Supplement: Supplementary file 1 [file ECE3-7-3507-s001.docx]
